# 

**Published:** 1902-11-08

**Authors:** 


NOTICE TO CORRESPONDENTS.

All MSS., Letters, Books for Review, and other matters intended for the Editor should be addressed The Editor, "The Hospital," The Hospital Building, 28 & 29 Southampton Street, Strand, W.C.

The Editor cannot undertake to return rejected MS., even when accompanied by stamped directed envelope.

**Notice to Advertisers and Subscribers.**

All Advertisements, Orders for Copies of The Hospital, and Business Communications should be addressed to **THE MANAGER, (Not to the Editor)**, The Hospital, 28 & 29 Southampton Street, Strand, London, W.C.

The Cost of Subscription, post free, is as follows.—Per week, 2½d.; per quarter, 2s. 8d.; per half-year, 5s. 3d.; per annum, 10s. 6d. Foreign:—Per annum, 15s. 2d., payable, in all cases, in advance.

**NOTICE.—Vol. XXXII. of "The Hospital" (April to September 1902) in handsome cloth binding will shortly be on sale at the office of this Journal, price, 7s. 6d. Cases for binding the Numbers contained in this Volume 1s. 6d. Index to Vol. XXXII., 2½d., post free.**

**The Monthly Part for November is now ready. Price 6d., by post 8d.**

### BOOKS RECEIVED.

Walter Scott Publishing Company.

"The Story of Life." By Ellice Hopkins.

Simpkin, Marshall, Hamilton, Kent and Co.

"The Record of a Child's Life." By Florence M. Hatton-Ellis

### The Student of Medicine.

### APPOINTMENTS.

**G. L. Bates**, M.R.C.S., L.R.C.P., L.D.S., appointed Dental Surgeon to the Royal Hospital School, Greenwich. **Sidney Bree**, M.R.C.S., L.R.C.P.Lond., appointed Certifying Factory Surgeon for the Manningtree District of the county of Essex. **A. W. Budden**, M.B., Ch.B.Vict., appointed Assistant Medical Officer to the West Derby Union Workhouse. **D. Crowe**, M.B.Dubl., B.Ch., appointed District Medical Officer of the Glandford Brigg Union. **George J. Dudley**, M.R.C.S., L.R.C.P.Lond., appointed Honorary Surgeon to the Stourbridge Dispensary. **A. O. M. Fehrsen**, M.R.C.S.Eng., L.R.C.P.Lond., appointed Assistant Medical Officer to the Birmingham Parish Infirmary. **Charles H. Fennell**, M.A., M.D.Oxon., M.R.C.P.Lond., appointed Assistant Medical Officer to Darenth Asylum. **W. D. Harmar**, M.A., M.B.Cantab., F.R.C.S., appointed Assistant Surgeon to the Metropolitan Hospital, Kingsland Road, N.E. **F. A. Knight**, M.D. (U.S.A.), appointed Clinical Assistant to the Chelsea Hospital for Women. **E. Graham Little**, B.A.CapeUniv., M.D., M.R.C.P.Lond., M.R.C.S.Eng., appointed Physician for Diseases of the Skin at St. Mary's Hospital. **J. O. Littlewood**, L.R.C.P.Lond., M.R.C.S.Eng., D.P.H., appointed Certifying Factory Surgeon for the Mansfield District of the County of Nottingham. **Robert William McCredie**, M.B., Ch.M.Syd., appointed Government Medical Officer and Vaccinator at Brewarrina, New South Wales. **Alexander N. McKelvey**, L.R.C.P.&S.I., appointed Assistant Medical Officer to the Lunatic Asylum, Auckland, New Zealand. **Charles G. Moffit**, M.R.C.S., L.R.C.P., D.P.H., Lond., appointed Junior Medical Officer in the Department of Lunacy, New South Wales. **W. S. Page**, M.B.Lond., M.R.C.S.Eng., appointed First Assistant Medical Officer to the St. Marylebone Infirmary. **Laurence C. Panting**, M.A., M.D. B.Ch.Oxon., appointed Honorary Medical Officer to the Royal Cornwall Infirmary, Truro. **W. C. Rigby**, M.B., Ch.B.Vict, appointed Certifying Factory Surgeon for the Adlington District of the County of Lancaster. **Mrs. Scharlieb**, M.D., M.S., appointed Physician for Diseases of Women, Royal Free Hospital. **James Swain**, M.S., M.D.Lond., F.R.C.S.Eng., appointed Honorary Surgeon to the Bristol Royal Infirmary. **Frederic R. P. Taylor**, M.D., B.S.Lond., appointed Medical Superintendent of the new East Sussex Asylum at Hellingly. **Frank B. Thornton**, M.B., B.S.Lond., etc., appointed an Honorary Physician to the Derbyshire Royal Infirmary. **H. N. Turner**, M.B., B.S.Glasg., appointed District Medical Officer of the Bourne Union. **Miss Ethel Vaughan**, M.D., B.S., appointed Assistant Physician for Diseases of Women, Royal Free Hospital. **Miss B. E. Walters**, M.B.Lond., appointed Assistant Medical Officer to the St. Pancras Workhouse. **R. Whittington**, B.A., M.B., B.Ch.Oxon., M.R.C.S.Eng., L.R.C.P.Lond., appointed Assistant Medical Officer to the Warneford Asylum, Oxford. **J. T. Williams**, M.B.Lond., L.R.C.P.Lond., appointed Second Assistant Medical Officer to the St. Marylebone Infirmary. **T. F. Williams**, M.B., M.S.Edin., appointed District Medical Officer of the Hawarden Union. **T. B. Young**, M.D.Brux. M.R.C.S.Eng., L.R.C.P.&S.Edin., appointed District Medical Officer of the Stourbridge Union.

### "The Hospital" Institutional Library.

"Hospitals and Asylums of the World." 4 Vols., with a Portfolio of Plans, complete £12 12s.

"Burdett's Hospitals and Charities, 1902." 5s.

"Cottage Hospitals, General, Fever, and Convalescent." 10s. 6d.

"Hospital Expenditure: The Commissariat." 2s. 6d.

"A Legal Handbook for the Use of Hospital Authorities." 2s. 6d.

"The Cottage Hospital Case Book or Register of Patients." 10s. and 12s. 6d.

"The Furnishing and Appliances of a Cottage Hospital." 6d.

All these are published by the Scientific Press, Ltd., and may be obtained through any bookseller, or direct from the publishers, 28 and 29 Southampton Street, Strand, London, W.C.

